# SARS-CoV CH.1.1 Variant: Genomic and Structural Insight

**DOI:** 10.3390/idr15030029

**Published:** 2023-05-24

**Authors:** Liliana Bazzani, Elena Imperia, Fabio Scarpa, Daria Sanna, Marco Casu, Alessandra Borsetti, Stefano Pascarella, Nicola Petrosillo, Eleonora Cella, Marta Giovanetti, Massimo Ciccozzi

**Affiliations:** 1 Sciences and Technologies for Sustainable Development and One Health, University Campus Bio-Medico of Rome, 00128 Rome, Italy; liliana.bazzani@alcampus.it; 2 Unit of Medical Statistics and Molecular Epidemiology, University Campus Bio-Medico of Rome, 00128 Rome, Italy; 3 Unit of Gastroenterology, Department of Medicine, University Campus Bio-Medico of Rome, 00128 Rome, Italy; 4 Department of Biomedical Sciences, University of Sassari, 07100 Sassari, Italy; 5 Department of Veterinary Medicine, University of Sassari, 07100 Sassari, Italy; 6 National HIV/AIDS Research Center (CNAIDS), National Institute of Health, 00161 Rome, Italy; 7 Department of Biochemical Sciences “A. Rossi Fanelli”, Sapienza University of Rome, 00185 Rome, Italy; 8 Infection Prevention and Control—Infectious Disease Service, Fondazione Policlinico Universitario Campus Bio-Medico, 00128 Rome, Italy; 9 Burnett School of Biomedical Sciences, University of Central Florida, Orlando, FL 32816, USA; 10 Instituto Rene Rachou Fundação Oswaldo Cruz, Belo Horizonte 30190-002, MG, Brazil

**Keywords:** CH.1.1 variant, genomic surveillance, SARS-CoV-2, mutational pattern profile

## Abstract

In early February 2023, the Omicron subvariant XBB.1.5, also known as “Kraken”, accounted for more than 44% of new COVID-19 cases worldwide, whereas a relatively new Omicron subvariant named CH.1.1, deemed “Orthrus”, accounted for less than 6% of new COVID-19 cases during the subsequent weeks. This emerging variant carries a mutation, L452R, previously observed in the highly pathogenic Delta and the highly transmissible BA.4 and BA.5 variants, necessitating a shift to active surveillance to assure adequate preparedness for likely future epidemic peaks. We provide a preliminary understanding of the global distribution of this emerging SARS-CoV-2 variant by combining genomic data with structural molecular modeling. In addition, we shield light on the number of specific point mutations in this lineage that may have functional significance, thereby increasing the risk of disease severity, vaccine resistance, and increased transmission. This variant shared about 73% of the mutations with Omicron-like strains. Our homology modeling analysis revealed that CH.1.1 may have a weakened interaction with ACE2 and that its electrostatic potential surface appears to be more positive than that of the reference ancestral virus. Finally, our phylogenetic analysis revealed that this likely-emerging variant was already cryptically circulating in European countries prior to its first detection, highlighting the importance of having access to whole genome sequences for detecting and controlling emerging viral strains.

## 1. Introduction

The Coronavirus disease 2019 (COVID-19) pandemic, caused by the Severe Acute Respiratory Syndrome Coronavirus 2 (SARS-CoV-2), continues to be a major public health issue. In February 2023, the WHO declared over 750 million confirmed cases and over 6.8 million deaths globally [1]. Since the onset of the pandemic in December 2019, variants of SARS-CoV-2 have emerged repeatedly. Some variants have spread worldwide and drove epidemic resurgences in many different regions. Several strains were identified as Variants of Concern (VOCs), including Alpha, Beta, Delta, Gamma, and Omicron since they exhibited increased transmissibility and/or immune evasion properties that threatened global efforts to control the pandemic [1,2,3,4,5]. More recently, novel Omicron sub-variants BA.2-like, BA.4-like, and BA.5-like lineages with novel defining point mutations were identified, which in turn appear to be critical for escape from immune responses generated against older variants [1]. In this respect, understanding the real-time evolution of SARS-CoV-2 is essential to control and ultimately end the pandemic. Current strategies, including the use of next-generation sequencing (NGS) on a large scale, appear to be a useful toolkit for virus variants detection [6]. Within the Omicron sub-variants, a newly emerged lineage, CH.1.1, first detected in Southeast Asia in early November 2022, has been moving public awareness [7]. It currently accounts for more than 33% of infections in some parts of the UK, 10% in the USA, 8% in Denmark, 6% in New Zealand, and 6% in Japan [7] CH.1.1, also called Orthrus, is causing alarm due to the presence of the L452R mutation in the S protein, which already appeared in Delta and BA.4/5 variants, likely able to increase its transmissibility [7]. In such a manner, understanding promptly the evolution of SARS-CoV-2 and its mutational profile appears to be crucial to apply effective prevention strategies. In this study, we performed a genome-based survey to assess genetic variability and phylogenetic and protein structural analysis with the aim of obtaining the epidemiological trajectory and dangers that CH.1.1 could represent worldwide.

## 2. Materials and Methods

All SARS-CoV-2 CH.1.1 whole genome sequences (n = 9726) and associated metadata available at the Global initiative on sharing all influenza data (GISAID-https://www.gisaid.org/, accessed on 18 March 2023) [8] were retrieved up to 28 February 2023. To ensure the quality of the data analyzed in this study and to guarantee the highest possible accuracy of the obtained results, only genomes >29,000 bp and <1% of ambiguities with a variant assignment provided by the Phylogenetic Assignment of Named Global Outbreak Lineages (PANGOLIN) [9] were selected. Genome sequences were aligned using the ViralMSA tool [10,11], and IQ-TREE2 [12] was used for phylogenetic analysis using the maximum likelihood approach. TreeTime [13] was used to transform this ML tree topology into a dated tree using a constant mean rate of 8.0 × 10^−4^ nucleotide substitutions per site per year after the exclusion of outlier sequences. The mutation pattern of the VOC was analyzed using the NextClade online tool [14].

## 3. Modeling

Homology models of BA.2.75 and CH.1.1 Spike RBDs were constructed using Modeller 10.3 [15], with the SARS-CoV-2 RBD in the PDB structure 6M0J as a template. The resulting models were visualized and analyzed using PyMOL v.2 [16]. To optimize the side chain conformation, FoldX 5.0’s “RepairPDB” [17] function was applied to the models.

To explore the conformational fluctuations and interactions, 100 homology models of the RBD and NTD domains were generated using Modeler. The refinement stage of homology modeling produced alternative models with varying conformational details, including side-chain rotamers. Each model was further optimized using the "RepairPDB" function in FoldX 5.0. Structural properties were calculated for all models, and average values with standard errors were determined. Net charges were predicted using PROPKA3 [18] at a reference pH of 7.0, though it may not reflect the physiological environment accurately.

The program APBS [19] was utilized to calculate the surface electrostatic potential, which was displayed as a two-dimensional projection using SURFMAP software [20]. SURFMAP implements molecular cartography to project the three-dimensional protein surface onto a two-dimensional plane, enabling the analysis and comparison of different physicochemical features across the protein surface.

Model structures of complexes between ACE2 and BA.2.75 and CH.1.1 RBDs were constructed using Modeler, using the SARS-CoV-2 complex reported in PDB structure 6M0J as a template. The interaction energy between Spike RBD and ACE2 was predicted using Foldx 5.0’s “AnalyseComplex” function and MM/GBSA (Molecular Mechanics/Generalized Born Surface Area) available on the HawkDock server [21]. Foldx 5.0 employs an empirical force field to describe various free energy terms, including electrostatic interactions, hydrogen bonds, desolvation, and van der Waals contacts. MM/GBSA HawkDock combines molecular mechanics calculations and continuum solvation methods to calculate binding free energies for macromolecules.

## 4. Results

**Figure 1:**
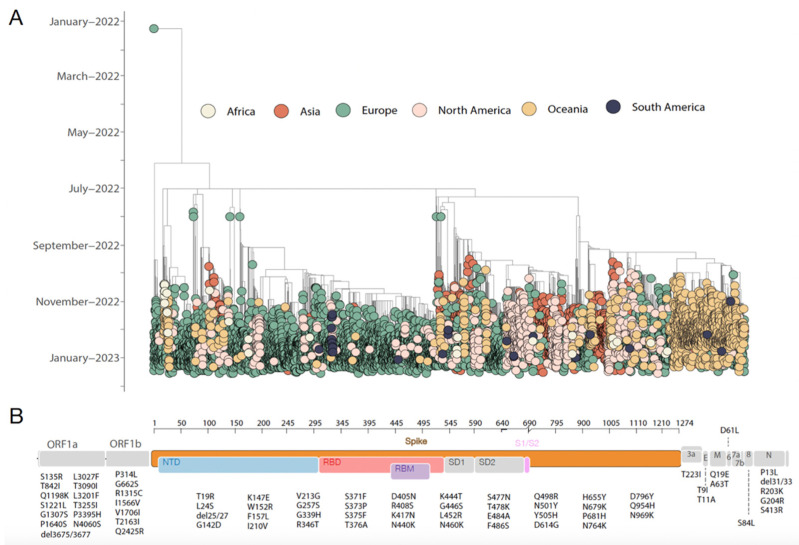
Spatial and temporal distribution of SARS-CoV-2 CH.1.1 strains worldwide. (**A**) Time-resolved maximum-likelihood tree of SARS-CoV-2 CH.1.1 whole genome sequences (n = 9726) collected up to 28 February 2023. The genomes are colored according to their isolation continent. (**B**) Genome map of the CH.1.1 lineage characteristic substitutions. Each gene is labeled, and the substitutions are placed at the bottom.

The recently described SARS-CoV-2 CH.1.1 variant, a descendant of the Omicron BA.2.75 VOC, was first detected in early July 2022 in India. By conducting a comprehensive phylogenetic reconstruction including all high-coverage CH.1.1 SARS-CoV-2 whole genome sequences available at GISAID up to 28 February 2022, we revealed that this likely-emerging variant was already cryptically circulating within European countries prior to its first detection, highlighting how the availability of whole genome sequences is pivotal to quickly detect emerging viral strains and implement effective control measures. Our analysis further revealed that the Orthrus variant, already widespread across the globe, is already isolated in all continents and is being detected already in at least 75 countries and 52 U.S. states [22]. Our time-stamped tree revealed a path of spreading already described for SARS-CoV-2, which underlines a massive international virus exchange responsible for outlining the rapid expansion of this novel strain all around the world (Figure 1A).

Currently, the CH.1.1. constitutes an estimated 10% or so of all the COVID-19 samples around the world [23]; according to recently released data (GISAID, 2023), it is most prevalent in Europe (64%), followed by Asia (14.4%), Oceania (12%), and North America (8.8%) while Africa and South America represented the less than 1% genomes.

Further, we analyzed the mutational profile to determine its lineage-defining mutations. The identified lineage harbored 70 substitutions that appear to be characteristic CH.1.1 substitutions interspersed in mostly the genome length (Figure 1B). CH.1.1 has a BA.2-like mutational profile as 51 of the substitutions highlighted are shared with BA.2 sub-lineages; similarly, it shared 51 and 50 mutations with BA.4 and BA.5, respectively. Out of these, thirty-nine mutations occurred in the spike region, 74% in the RBD portion (n = 29), and 26% in NTD (n = 10). The remaining 19 mutations (19/70, 27%) not shared with other Omicrons sublineages were mainly in the spike protein (60%, n = 5 substitution in NTD and n = 6 in RDB), and the remaining were in ORF1a-b (35%, n = 7) and one mutation in the envelope protein (5%).

**Figure 2:**
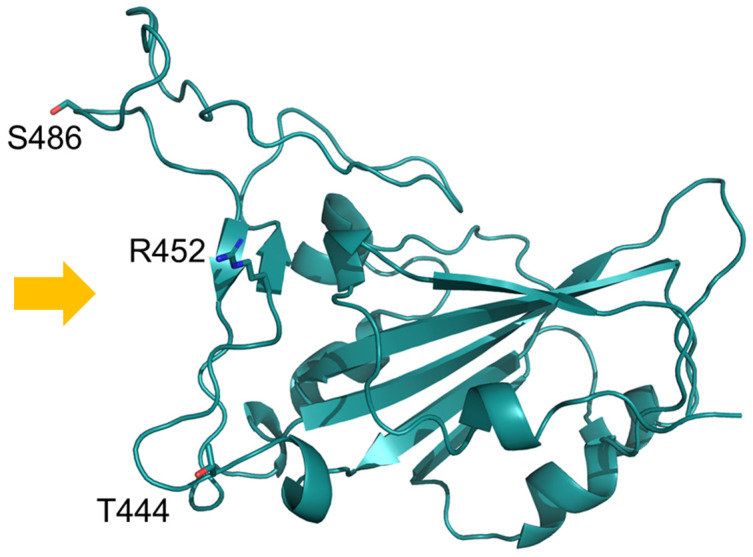
Model of CH.1.1 RBD displayed as a ribbon model. Mutant residues unique to CH.1.1 with respect to BA.2.75 are shown with labeled stick models. Orange arrows indicate the side interacting with ACE2.

Figure 2 reports the mapping of mutations unique to CH.1.1 RBD compared to BA.2.75. The mutation F486S occurs at the ACE2 interface and removes hydrophobic interactions with ACE2 L79, M82, and Y83 (Supplementary Figure S1), as it is also observed within the XBB and XBB.1 variants [24].

**Figure 3:**
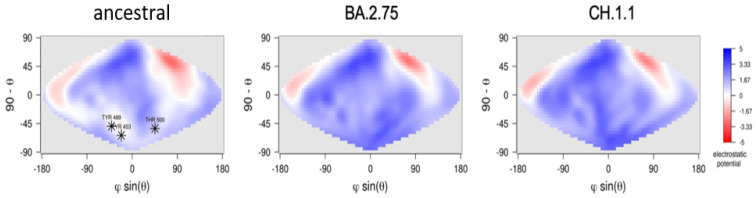
Projection on a two-dimensional map of the electrostatic potential surface of RBDs of the three viruses. The color scale is reported aside from the ancestral strain map. Electrostatic potential values are expressed as kT/e units. Map axes report the projected polar coordinates of the domains. Asterisks in the ancestral strain map indicate the position of the three residues, T453, Y489, and T500, belonging to the interface with ACE2.

Two additional CH.1.1 mutant sites (R452 and T444) appear to be localized near the ACE2 interface, but our analysis revealed they are not directly interacting with its residues. The net charge of BA.2.75 and CH.1.1 RBDs appear to be similar: 5.45 ± 0.02 and 5.43 ± 0.02, respectively. The electrostatic potential surface displays a similar distribution in the two variants and appears to be more positive than in reference to the ancenstral virus (Figure 3). NTDs residues of BA.2.75 and CH.1.1. appear to be identical and, for this reason, were not considered further.

Spike RBD-ACE2 interaction energy prediction (Table 1) suggests that CH.1.1 presented a somewhat weaker interaction, likely due to the lack of F486 as observed in XBB and XBB.1 [25].

The method implemented in MM/GBSA can also assess the energetic contribution of single residues to the thermodynamic stability of the ACE-RBD interface. The residues in CH.1.1 and BA.2.75 that are predicted to contribute more than 2.0 Kcal/mol to the interface stability are Y714, F456, N487, Y489, R498, Y501, and H505. Among these residues, R498 and H505 give the highest contribution.

## 5. Discussion

As SARS-CoV-2 continues to spread and cause diseases, emerging variants of the virus are being identified around the globe. The persisting challenges of SARS-CoV-2 to the international public health system have elicited concerns related to the capacity of some strains to be able to enhance viral transmissibility or being associated with differential clinical outcomes and viral escape. In this respect, the World Health Organization (WHO) has designated some variants as “Variants of Concern (VOCs)” or “Variants of Interest (VOIs)” because of their ability to significantly change the virus’ pathogenesis.

In early July 2022, a descendant of the Omicron BA.2 sublineage carrying a critical mutation, the L452R in the spike glycoprotein, which was predicted to influence antibody neutralization and spike function, was first identified.

In this study combining genomic data with a structural molecular modeling approach, we describe the mutational pattern profile and early transmission dynamics of the so-called Orthrus lineage, highlighting the rapid spread in regions with high levels of population immunity.

The February 2023 COVID-19 epidemiological report from the World Health Organization (WHO) lists Orthrus among the top three most prevalent variants in Europe, clocking in at 12.3%, slightly behind BQ.1 at 13% and BQ.1.1 at 31.3% [1].

Our phylogenetic reconstruction revealed that this emerging variant was cryptically circulating during a period of six months within European countries prior to its first detection in the Asian continent. Although the heterogeneous and uneven sampling that we have observed could create biased results in the epidemic reconstructions through phylogenesis and to properly address it, a subsampling that heeds and reflects global reported case counts should be considered. Our time-stamped tree additionally picked multiple introduction events all over the world, highlighting how critical it will be to integrate genome sequence with human mobility data to promptly reconstruct and track the transmission dynamics of those emerging strains. Continuous exchange among the countries involved was observed, suggesting how dynamic is the variant spread, as we observed for the other lineages previously.

To understand the functional properties of the single point mutations carried by this variant, we further performed a mutational pattern analysis. This emerging lineage carries 39 mutations in the spike protein (55.7%) that may have functional importance, in addition to several mutations seen in the Delta variant related to increased risk of disease severity, risk of vaccine escape, and higher transmissibility. It is noteworthy that the remaining 44.3% of the mutations are dispersed throughout the genome. The virus attempts to develop new mutations beyond the spike protein as it tries to evade the immune response of T lymphocytes by introducing mutations in the N protein and the ORF proteins. In this respect, recently released data pointed out the continued waning of 3-dose mRNA booster efficacy against newly emerging Omicron subvariants. This effect can be partially saved by the administration of a bivalent booster, through escape by some subvariants, including the CH.1.1. In fact, the CH.1.1 variant exhibited almost complete escape from neutralization by triple-vaccinated or BA.4/5 infection sera, warranting continued surveillance and further analyses [25].

In addition to that, our structural modeling prediction revealed that BA.2.75 and CH.1.1 share identical NTDs and display RBDs with similar characteristics. CH.1.1 may have a weaker interaction with ACE2, as F486S showed to hinder the binding, as is already happening with XBB.1.x. However, it cannot be excluded that some of the CH.1.1 specific point mutations might be able to alter the interaction with other molecular components or influence intramolecular communication with the NTD region [26]. Overall, no significant difference between CH.1.1 and its parent BA.2.75 has been detected using the sole basis of structural analysis. Furthermore, the residues that contribute most to the stability of the ACE-RBD interface are conserved in the two variants CH1.1 and BA.2.75.

This variant is spread almost worldwide, in at least 75 different countries, and even if its prevalence is still marginal, it has a unique combination of mutations in the spike protein compared to other Omicron subvariants. However, it is important to note that this variant is still being studied, and there is currently limited information available about its transmissibility, severity, and immune evasion capabilities.

In conclusion, our results point out how crucial it is to limit the spread of SARS-CoV-2 variants, including Omicron descendent lineages since these variants carry a constellation of mutations able to increase their prevalence or signs of growth rate advantage in some countries relative to other circulating variants, and additional amino acid changes that are known or suspected to confer a fitness advantage. Reducing transmission through established and proven disease control methods/measures, as well as avoiding introductions into animal populations, are crucial aspects of the global strategy to reduce the occurrence of mutations that have negative public health implications.

## Figures and Tables

**Table 1:** Predicted interaction energy between ACE2 and RBD expressed in kcal/mol.

Method	BA.2.75	CH.1.1
FoldX 5.0	−6.19 ± 0.32	−3.51 ± 0.30
MM/GBSA	−62.7 ± 0.4	−61.8 ± 0.8

## Data Availability

Genomes analyzed in the present study were taken from the GSAID database and are available at https://gisaid.org/, accessed on 18 March 2023.

## Supplementary Materials

The following supporting information can be downloaded at: https://www.mdpi.com/article/10.3390/idr15030029/s1, Figure S1: Comparison of complex between ACE2 (orange) and RBD (deep-teal) represented as cartoon models. The side chains of RBD position 486 are displayed along with ACE2 interacting residues as labelled stick models.
