# Supplementary figures and images for: SARS-CoV CH.1.1 Variant: Genomic and Structural Insight
Source: Infect Dis Rep. 2023 May 24;15(3):292–8. doi: 10.3390/idr15030029 (PMC10298543; doi:10.3390/idr15030029)

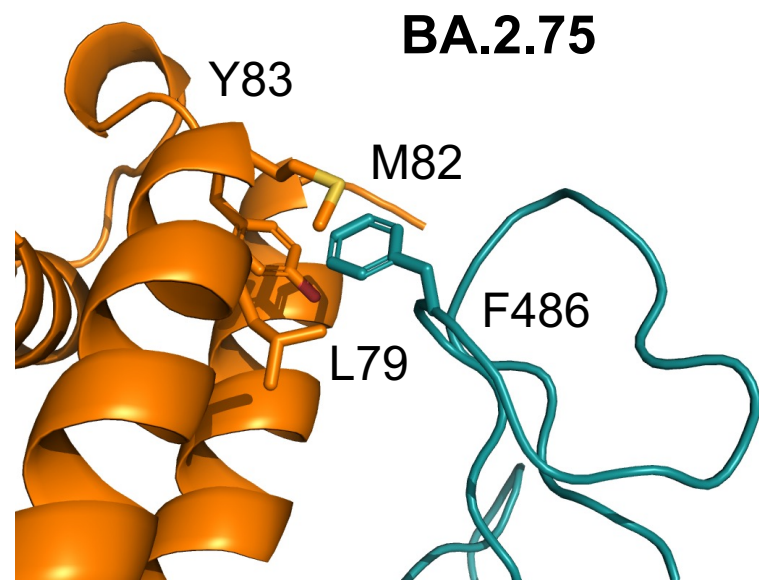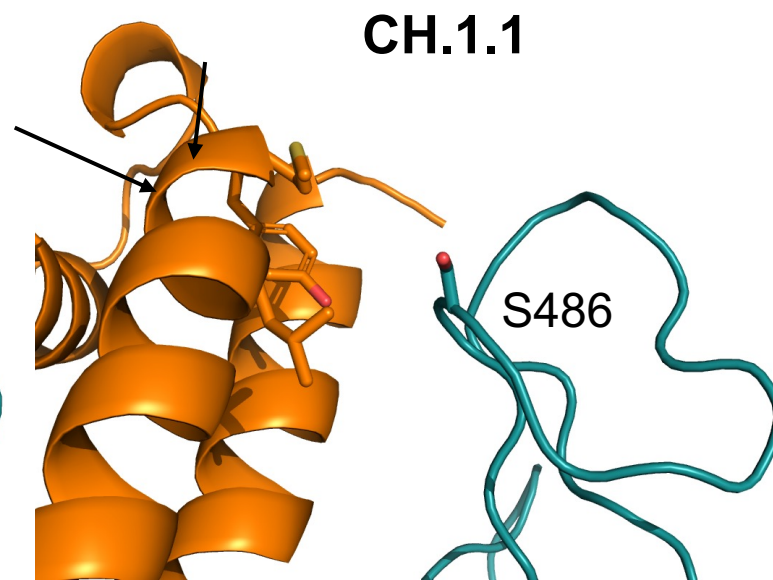

Supplement: Supplementary file 1 [file idr-15-00029-s001.zip › Figure_S1.pdf]
